# TRIF Is Required for TLR4 Mediated Adjuvant Effects on T Cell Clonal Expansion

**DOI:** 10.1371/journal.pone.0056855

**Published:** 2013-02-15

**Authors:** Siva K. Gandhapudi, Paula M. Chilton, Thomas C. Mitchell

**Affiliations:** 1 Institute for Cellular Therapeutics, University of Louisville School of Medicine, Louisville, Kentucky, United States of America; 2 Department of Microbiology and Immunology, University of Louisville School of Medicine, Louisville, Kentucky, United States of America; Oklahoma Medical Research Foundation, United States of America

## Abstract

Toll like receptor 4 (TLR4) is an important pattern recognition receptor with the ability to drive potent innate immune responses and also to modulate adaptive immune responses needed for long term protection. Activation of TLR4 by its ligands is mediated by engagement of the adapter proteins MyD88 (myeloid differentiation factor 88) and TRIF (Toll-interleukin 1 receptor domain-containing adapter inducing interferon-beta). Previously, we showed that TRIF, but not MyD88, plays an important role in allowing TLR4 agonists to adjuvant early T cell responses. In this study, we investigated the T cell priming events that are regulated specifically by the TRIF signaling branch of TLR4. We found that TRIF deficiency prevented the TLR4 agonist lipid A from enhancing T cell proliferation and survival in an adoptive transfer model of T cell priming. TRIF deficient DC showed defective maturation as evidenced by their failure to upregulate co-stimulatory molecules in response to lipid A stimulation. Importantly, TRIF alone caused CD86 and CD40 upregulation on splenic DC, but both TRIF and MyD88 were required for CD80 upregulation. The impairment of T cell adjuvant effects and defective DC maturation in TRIF ^lps/lps^ mice after TLR4 stimulation was mainly due to loss of type I IFN production, indicating that type I interferons are central to TLR4's adjuvant effects. These results are useful for the continued development of TLR4 based vaccine adjuvants that avoid inflammatory risks while retaining beneficial immune response.

## Introduction

Toll like receptor 4 (TLR4) is a component of an evolutionarily conserved pattern recognition receptor protein complex that evolved to recognize microbial lipopolysaccharides (LPS) as well as several host derived damage associated molecules such as heat shock proteins, and high mobility group proteins HMGB1 and HMGN1[1], [2], [3]. TLR4 receptors are type I transmembrane proteins containing extracellular leucine rich repeats and intracellular TIR signal domains [4], and are expressed on a variety of host immune and non-immune cells. Activation of TLR4 is driven by the engagement of two important adaptor protein molecules, MyD88 (myeloid differentiation factor 88) and TRIF (Toll-interleukin 1 receptor domain-containing adapter inducing interferon-beta) [3], [5], [6], [7]. Engagement of the MyD88-dependent branch rapidly leads to activation of NFκB and MAPK, which drive proinflammatory gene expression. Several minutes later, engagement of the TRIF dependent branch via the endocytic pathway activates interferon regulatory factors and ‘late’ NFκB [6], [8]. TLR4 stimulation thus plays a role in initiation of rapid innate immune responses, as well as an important role in modulation of adaptive immune responses to eliminate the pathogen and to mount protective memory immune responses [9].

Because TLR4 can stimulate both innate and adaptive immune responses to combat microbial infections, it has become an attractive target for pharmacologic manipulations aimed at vaccine adjuvant development [10], [11], [12]. Specifically, the TLR4 agonist monophosphoryl lipid A (MPL®), is a low toxicity derivative of LPS from the *Salmonella minnesota* strain Re595 that has been recently approved for use in vaccines against human pathogens such as human papilloma virus and hepatitis B virus [10]. Several clinical studies on the activity of MPL® have shown that it is safe adjuvant for use in prophylactic vaccines [13], [14]. However, due to the toxic nature of its parent compound, LPS, and the technical challenges associated with purification of MPL, focus has shifted to next-generation synthetic derivatives that may possess similar or better adjuvant properties with even better safety profiles [15].

Currently approved vaccines function primarily by establishing high affinity antibody responses, which require T cell help for isotype switching and affinity maturation. Hence, a critically important component of the adjuvant effects through TLR4 is at the level of T cell priming upon immunization. Unlike some TLRs, TLR4-mediated adjuvant effects on T cell priming occur indirectly through activation of antigen-presenting cells (APC) [16]. TLR4 engagement causes APC maturation leading to the upregulation of MHC and co-stimulatory molecules [8], [17], [18], and to the production of chemokines and cytokines [7]. Each of these APC activities can modulate T cell clonal expansion, effector function and differentiation [19], [20], [21]. T cell clonal expansion immediately following antigen stimulation is a critical step that can influence downstream T cell responses including differentiation and memory establishment [22]. Hence, a better understanding of the mechanistic details of TLR4 signaling events needed for T cell priming is necessary for identifying and developing compounds that can potentially uncouple the favorable adaptive immune responses from the unfavorable or unnecessary pro-inflammatory responses.

In an earlier study we showed that a potency-adjusted dose of a generic version of monophosphoryl lipid A (MPLA) induced weak MyD88-dependent cytokine production compared to LPS, while the same dose was as effective in adjuvanting T cell clonal expansion as its parent LPS molecule [23]. Furthermore, adjuvant effects on T cells mediated by either MPLA or LPS were markedly reduced in mice lacking functional TRIF, but not the MyD88 adapter protein. Although the underlying mechanism was not defined, this earlier study showed the importance of functional TRIF in mediating TLR4 induced adjuvant effects on T cell clonal expansion. In the current study, we extended our investigation further to understand the aspects of T cell priming events that are influenced by TRIF when adjuvanted with the classical TLR4 agonist, lipid A. We used lipid A, the minimal TLR4 activating LPS moiety, to study TLR4 activation because LPS preparations are more heterogeneous in nature and often have contaminants that potentially confound the TRIF signal effects by influencing dendritic cell maturation and T cell priming [24], [25].

Type I IFNs are important TRIF dependent cytokines produced during TLR4 stimulation. Earlier studies have shown that type I IFNs produced after TLR4 stimulation can cause APC maturation in vitro [17], [18]. However, the role of type I IFNs in TLR4-mediated adjuvant effects on T cell priming is not fully defined. Therefore, we used type I IFN receptor blocking antibodies (MAR1-5A3) [26] and recombinant type I IFN substitution models to study the need for type I IFN signaling in TLR4 stimulation. This approach has advantages over type I IFN receptor deficient (IFNAR1^−/−^) mice, which exhibit abnormalities in macrophage and dendritic cell functions [27], [28] that can confound interpretation of the effects of TLR4 induced type I IFN on T cell priming.

Our results show that TRIF plays a crucial role in adjuvanting T cell clonal expansion upon TLR4 stimulation by influencing dendritic cell maturation, and adjuvanted T cell proliferation and survival. Furthermore, we show that TRIF dependent type I IFN is a central underlying mechanism for TLR4-mediated adjuvant effects on T cell priming by TLR4 agonists.

## Materials and Methods

### Ethics Statement

All animals used in this project were housed and bred in a specific pathogen-free animal housing facility and all animal protocols used in this study were reviewed and approved by the University of Louisville Institutional Animal Care and Use Committee (IACUC No. 10005) following the National Institutes of Health animal care guidelines.

### Mice

Six to eight week old C57BL/6 mice, TRIF ^lps/lps^ breeder mice, and CXCL10^−/−^ breeder mice were purchased from Jackson laboratories (Bar harbor, ME). MyD88^−/−^ breeder mice were a kind gift from Shizuo Akira (via Ross Kedl, 3 M Corporation, Minnesota, MN). OTI.SJL (B6.SJL CD45a (Ly5a)/NAi (B6.SJL), transgenic mice with T cells expressing TCR that is specific to ovalbumin peptide 257–264 presented in the context of MHC I (K^b^) [29] and OTII.SJL (B6.SJL CD45a (Ly5a)/NAi (B6.SJL) transgenic mice with T cells expressing TCR that is specific for ovalbumin-peptide 323–339 presented in the context of MHC II (I-A^b^) [30] were originally purchased from Taconic Farms (Germantown, NY) and bred at the Institute of Cellular Therapeutics, University of Louisville.

### Reagents

TLR4 agonist lipid A (*Salmonella minnesota* Re 595) was purchased from ENZO LIFE Sciences Inc. (Farmingdale, NY). Endotoxin-free ovalbumin peptide 323–339 (OVA_323–339_; polypeptide sequence – ISQAVHAAHAEINEAGR) and ovalbumin peptide 257–264 (SIINFEKL) were obtained from (CPC Scientific Inc. Sunnyvale, CA). Recombinant mouse interferon β (IFN-β) was purchased from PBL interferon source (Piscataway, NJ). Anti-mouse IFNRα/β blocking mAb (MAR1-5A3) was purchased from Leinco Technologies (St. Louis, MO). Mouse monoclonal antibodies (mAb) for flow cytometric analysis were purchased from BD Pharmingen (San Diego, CA) and eBioscience (San Diego, CA). For lipid A adjuvant analysis fluorescein isothiocyanate (FITC) conjugated CD45.2, phycoerythrin (PE) conjugated CD8, peridinin-chlorophyll-protein complex (PerCP) CD4, and allophycocyanin (APC) conjugated CD45.1 were used to enumerate and analyze donor and recipient T cell populations. For analysis of APC activation, anti- mouse mAb specific for CD4, CD80, CD86, CD40 B220, CD11c, CD11b, CD8, and CD3 and CD19 were used. The isotype controls used for dendritic cells and macrophage activation markers were PE conjugated anti-hamster IgG_2_κ for CD80 and PE conjugated anti-rat IgG_2_aκ for CD86 and CD40.

### Adoptive transfer and activation of T cells in vivo

Spleens from OTI.SJL and OTII.SJL mice were aseptically removed and processed by passing through 70 µm cell strainers to obtain single cell suspensions. The cell suspensions were washed and suspended in HBSS at concentrations 1×10^5^ CD8^+^ OT I and 1.5×10^5^ CD4^+^OTII cells per 0.2 ml for transfer into recipient mice by intravenous tail injection. For experiments using higher cell concentrations 1×10^6^ CD8^+^ OT I and 1.5×10^6^ CD4^+^OTII cells per 0.2 ml were used. For cell division tests, OTI and OTII cells 4×10^7^ spleen cells/ml of HBSS were mixed with an equal volume of HBSS containing 5 µM cell Trace™ CFSE dye (Molecular Probes, Grand Island, NY) and were incubated at room temperature in dark for 15 min. Following the incubation, cells were washed twice with HBSS before re-suspending at the required concentrations for adoptive transfers. Forty-eight hours after adoptive transfer, the recipient mice were immunized via i.v. injections (200 µl injections) or through subcutaneous hock injections (50 µl injections split between two hind limbs) with HBSS (No Ag) or antigen alone (Ag; 10 µg SIINFEKL and 50 µg OVA_323–339_) or antigen and adjuvant Lipid A (Ag+Adj; 10 µg lipid A/mouse). At peak clonal expansion, the spleens and/or draining lymph nodes from the treated mice were removed and processed to obtain single cell suspensions for further studies.

### In vivo IFN-β treatment

To study the adjuvant effects of IFN-β, mice were injected subcutaneously in the hock region with 3 separate doses (30,000 IU/dose) of recombinant mouse IFN-β in 50 µl HBSS at 6, 12 and 18 hr after antigen injection.

### Splenic DC preparation, labeling and flow cytometric analysis

Harvested spleens were injected with 1 ml of collagenase medium containing 1 mg/ml collagenase IV (Worthington Biochemical Corporation, Lakewood, NJ), 10 µg/ml Dnase I (Roche Diagnostics, Indianapolis, IN), 2% FBS in RPMI 1640, the spleen was chopped into small fragments and incubated at 37°C for 30 min in 4.5 ml of collagenase medium. EDTA, 0.5 ml of 0.1 M, solution was added to the cultures in the last 5 min of the incubation to foster dissociation of T cell-APC conjugates. The entire culture was then passed through a 100 µm cell strainer, washed twice with HBSS and suspended at 10×10^7^ cells/ml of staining buffer containing 2% FBS and 0.02% Na_3_N in HBSS. 100 µl of cells suspensions were then labeled with antibody cocktail containing flurochrome conjugated anti-mouse CD3, CD19, CD4, CD8, B220, CD11c, CD11b and anti-mouse CD86 or CD80 or CD40. Labeled cells were then washed with HBSS, fixed with 2% formaldehyde (Polyscience Inc, Warrington, PA) and analyzed using a BD LSRII flow cytometer. Co-stimulatory molecule upregulation on different DC and CD11b^+^ subsets in spleen were analyzed using the appropriate gating strategy using Flow Jo software.

### Calculation of average number of cell divisions

The average number of T cells divisions following activation was estimated using methods described previously [31]. Briefly, CFSE dilution profiles of activated T cells were divided into successive bins corresponding to the number of divisions. The average number of divisions undergone by T cells was then estimated as the sum of the percentages of T cells in each bin multiplied by the division number of that bin. The zero division peak was estimated using the CFSE profile of the T cells isolated from a control group of mice at the time of immunization.

### Ex vivo adjuvant induced T cell survival assay

To analyze adjuvant induced T cell survival, 5×10^6^ splenocytes obtained from the immunized mice at peak clonal expansion phase were suspended in 1 ml of serum free RPMI medium and cultured in 5 ml polystyrene tubes kept in humidified incubators set at 37°C and 5% [CO_2_]. The cultures were processed and analyzed for the survival percentages at 9 or 18 h following incubation using live/dead gating methods described previously [32], which also shows equivalence to PI/Annexin staining.

### Statistical analysis

Statistical significance between variables was determined using two-way ANOVA and p values were estimated using the Bonferonni method in Graphpad Prism ® software.

## Results

### Functional TRIF is required for TLR4 adjuvant effects on T cell clonal expansion

**Figure 1:**
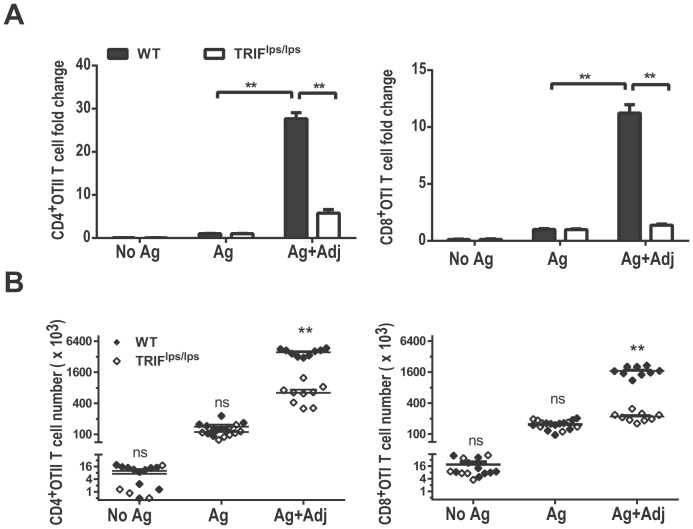
TLR4 mediated adjuvant effects require functional TRIF signal. OTI and OTII T cells were activated in WT or TRIF^lps/lps^ recipients with i.v. injections containing antigen alone (Ag) or antigen plus lipid A (Ag+Adj), or saline (No Ag). Spleens from recipient mice were harvested 96 h after immunization, and B) CD8^+^OTI and CD4^+^OTII cells were enumerated as described in methods and A) adjuvant effects on T cell clonal expansion were measured by calculating the fold-increase in total OTI or OTII T cell numbers in adjuvanted mice vs. mice given Ag alone. Results are from three independent experiments combining 3 replicates in each experiment (n = 9). **;P value<0.001 between Wt or TRIF^lps/lps^ and ns; not significant.

We previously demonstrated that TRIF-dependent, but not MyD88-dependent, signals are required for the LPS induced adjuvant effects on early T cell clonal expansion [23]. To confirm the importance of TRIF in TLR4 mediated increases in T cell clonal expansion, adoptively transferred CD8^+^ OTI and CD4^+^ OTII transgenic T cells were activated by treating C57BL/6 (WT) or TRIF^lps/lps^ recipients with peptide antigens alone or with lipid A, and peak clonal expansion was measured 96 h after immunization. As expected, the presence of lipid A during T cell priming significantly increased the peak burst size of both OTII and OTI T cells in WT recipients by an average of 27 and 11 fold, respectively, compared to non-adjuvanted recipients (Figure 1A). We found no apparent requirement for TRIF in the limited T cell clonal expansion that occurs in the absence of adjuvant, as evidenced by similar antigen stimulated OTI and OTII T cell expansion in WT vs. TRIF^lps/lps^ recipients (Figure 1B). TRIF deficiency however, abrogated the adjuvant effect of lipid A on OTI T cells and markedly reduced that for OTII T cells. This pattern was similar to our earlier study with LPS [23], although the requirement for TRIF in adjuvanting CD4^+^T cell responses was more severe when using lipid A as a TLR4 agonist. Thus these results clearly reinforce our earlier observation that TRIF-mediated signaling events are critical for TLR4 agonists to adjuvant CD4^+^ and CD8^+^ T cell clonal expansion early during the primary immune response.

### TRIF deficient dendritic cells do not upregulate co-stimulatory molecules upon lipid A activation in vivo

**Figure 2:**
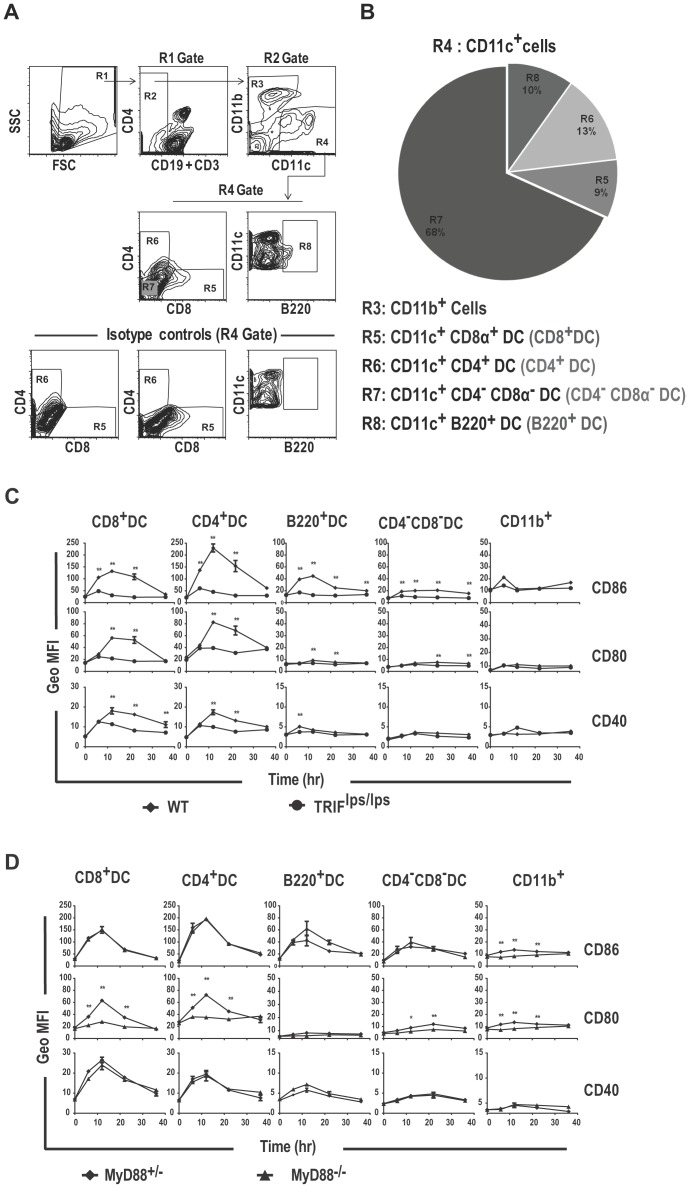
TLR4-mediated upregulation of co-stimulatory molecules on splenic DC subsets in vivo occurs in a TRIF dependent manner. Spleens from WT, TRIF^lps/lps^, MyD88^−/+^ and MyD88^−/−^ mice that had been injected with Lipid A (10 µg/mouse) or with saline were harvested after 6, 12, 22, or 36 hr and stained with flurochrome conjugated antibodies. Flow cytometric gating (A) was used to identify CD11c^+^, CD11b^dim/neg^ CD8α^+^ (CD8^+^ DC), CD11c^+^, CD11b^dim/neg^ CD8α^−^ CD4^+^ (CD4^+^DC), CD11c^+^, CD11b^dim/neg^ CD8α^−^ CD4^−^ (DNDC), CD11c^hi^, CD11b^dim/neg^ B220^+^ (B220^+^DC), and CD11c ^−^, CD11b^+^ (CD11b^+^ cells). Percentages of various DC populations within the CD11c^+^ compartment of untreated WT mice are shown (B). Expression of CD86, CD80 and CD40 on activated dendritic cell subsets from TRIF^lps/lps^ vs. WT (C) or from MyD88^−/+^ vs. MyD88^−/−^ mice (D) are represented as a time course (hr) showing the geometric mean fluorescence intensity on the indicated cell population. The data are representative of three independent experiments with 3 replicate mice each. Statistical significance between the treatments at each time point is estimated by two-way ANOVA. **; p-value≤0.01 and *; p-value≤0.05 confidence interval.

Mature DC play an essential role in priming naïve T cells to undergo clonal expansion by providing the necessary T cell activation and co-stimulatory signals. Because TLRs are potent inducers of DC maturation, we next investigated whether TRIF is required for DC maturation in response to TLR4 activation in vivo. The mouse splenic DC pool is a heterogeneous population consisting primarily of CD8^+^DC (CD11c^+^ CD11b^−^ CD8^+^) located in T cell rich regions, CD4^+^ DC (CD11c^+^ CD11b^−^ CD4^+^) and CD4^−^CD8^−^ DC (DNDC) (CD11c^+^CD11b^−^ CD4^−^CD8^−^) located in marginal zone regions, and plasmacytoid DC (B220^+^DC) (CD11c^+^ CD11b^−^ B220^+^) mainly located in the T cell rich, and to a lesser extent, in the marginal zone region [33], [34], [35]. We hypothesized that because these distinct DC subsets were known to differ in their expression of TLR [36], [37] stimulation via TLR4 could differentially affect DC subsets. To test this, spleen cells from WT, TRIF^lps/lps^, MyD88^+/−^, and MyD88^−/−^ mice that were injected with lipid A and harvested after 6, 12, 22, or 36 h and processed as described in Methods. We then gated on different dendritic populations in the spleen as shown in Figure 2A. After defining the relative frequencies of the DC populations at time 0 h (Figure 2B), we monitored the maturation of each of these subpopulations in WT vs. TRIF ^lps/lps^ mice after in vivo lipid A treatment based on expression levels of co-stimulatory molecules (Figure 2C). Among the different splenic DC subsets investigated, B220^+^ DC and DNDC showed the least response to the TLR4 activation signals in terms of upregulating CD86, CD80, and CD40 expression (Figure 2C), while CD8^+^ DC and CD4^+^ DC showed the biggest changes in the expression of these co-stimulatory molecules. In WT mice, TLR4 stimulation significantly increased the expression of CD86, CD80 and CD40 on CD8^+^ and CD4^+^ DC to a maximum within the first 12 h, which was maintained through at least 22 h (Figure 2C). In contrast, TLR4 stimulation in TRIF ^lps/lps^ mice caused only weak upregulation of CD86 in the first 6 h that dropped to levels comparable to unstimulated DCs thereafter. Although lipid A induced upregulation of CD80 and CD40 in TRIF^lps/lps^ mice was often similar to WT during the first 6 h, their expression levels beyond 6 h were significantly lower compared to WT mice.

An earlier study by Shen et.al [18] showed that LPS requires both MyD88 and TRIF signals to upregulate co-stimulatory molecules on DC as identified by CD11c expression. However, when we investigated the contribution of MyD88 signaling using the highly purified TLR4 agonist lipid A in more DC subsets, we found in both CD8^+^ and CD4^+^ DC that upregulation of CD86 and CD40 was independent of MyD88 (Figure 2D). On the other hand, upregulation of CD80 in response to lipid A was significantly lower in the absence of MyD88. Thus, our results indicate that TRIF and MyD88 play distinct roles in upregulating different co-stimulatory molecules on DC in vivo after TLR4 activation. Although the upregulation of CD80 required both TRIF and MyD88, the upregulation of CD86 and CD40 was solely dependent on TRIF- associated signaling events in our experiments.

### TLR4 adjuvanted T cells undergo fewer divisions in the absence of TRIF signaling

**Figure 3:**
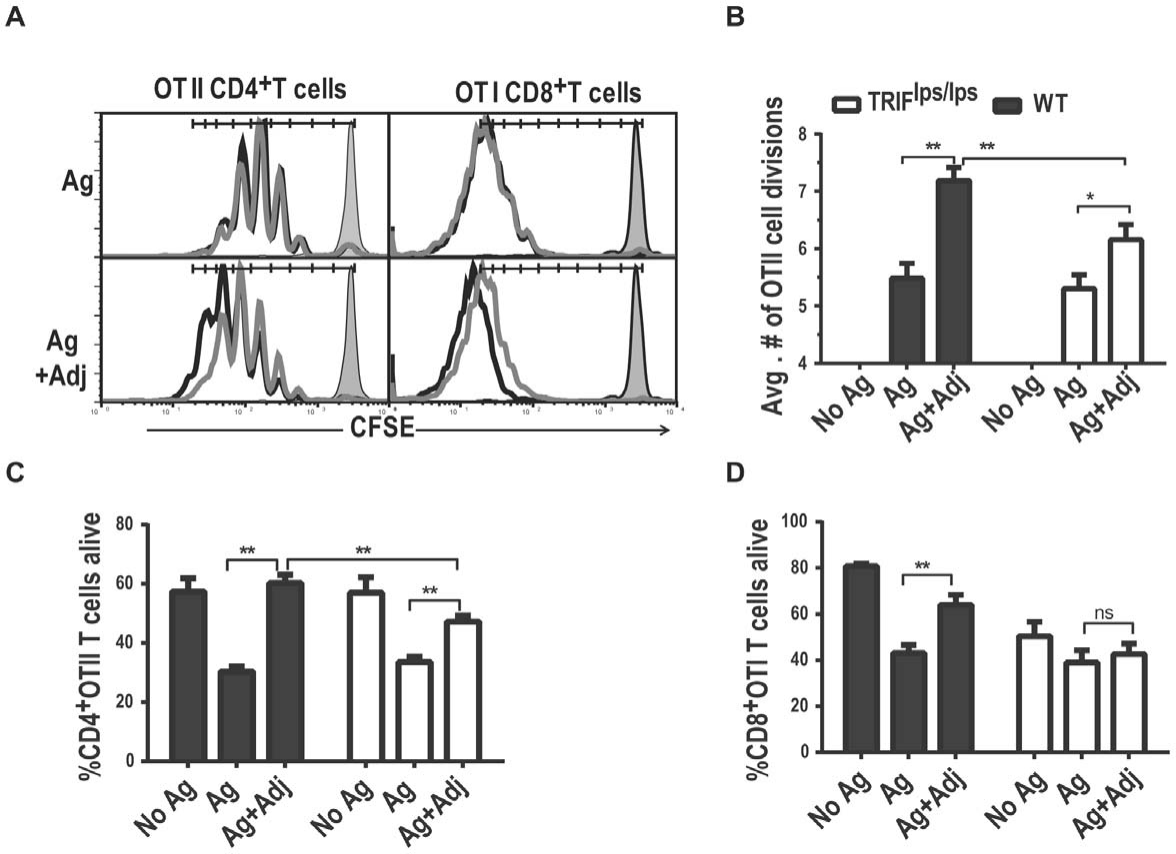
Lipid A adjuvanted T cells in TRIF^lps/lps^ recipients have impaired T cell proliferation, and survival ex vivo under growth factor deprived conditions. CFSE labeled OTI and OTII cells were activated in WT and TRIF^lps/lps^ recipients with antigen alone (Ag), antigen plus lipid A (Ag+Adj), or saline (No Ag) and harvested for flow cytometric analysis 96 h later. A) Histograms show the CFSE profiles of representative replicates after each treatment (black lines, WT; gray lines, TRIF^lps/lps^; solid histogram, unstimulated cells obtained at the time of immunization).B) The average number of OTII cell divisions after each treatment were calculated as described in Methods. Results shown are the combined average ± SEM of 3 replicates from three independent experiments (n = 9). C–D) TLR4-induced survival effects on the proliferating T cells were estimated by placing the harvested splenocytes in ex vivo culture under growth factor restricted conditions and testing for viable cells after 9 h (CD8^+^ OTI cells (C)) or 18 h (CD4^+^OTII cells (D)). Data represented are the averages from all 3 replicates from 3 independent experiments. Statistical significance between the treatments was by two-way ANOVA. **; p-value≤0.01 and *; p-value≤0.05.

The clonal burst size of activated T cells is influenced by a number of factors including the proportion of antigen specific T cells that become activated and the extent to which the T cells divide and survive during clonal expansion. To test whether any of these factors are most dependent on TRIF-mediated signaling, we labeled OTI and OTII T cells with CFSE and transferred them into WT or TRIF^lps/lps^ recipients prior to immunization with antigen alone or with antigen and lipid A. Antigen stimulation caused OTII T cells to undergo relatively robust proliferation compared to non-treated mice in both WT and TRIF ^lps/lps^ recipients (Figure 3A), with the number of cell divisions averaging 5.4±0.2 and 5.3±0.2 WT and TRIF ^lps/lps^ mice, respectively (Figure 3B). Consistent with earlier reports [38], lipid A as adjuvant significantly increased the number of OTII T cell divisions by an average of 1.7±0.2 divisions in WT recipients compared to antigen alone control treatment. Interestingly, in TRIF^lps/lps^ mice adjuvant treatment caused only a modest increase, 0.8±0.2 divisions, over non-adjuvanted treatments, indicating that TLR4 stimulation has a suboptimal response in terms of increasing T cell proliferation in the absence of TRIF-dependent signaling. Similar studies on OTI cell division were inconclusive. Even with transfer of high cell numbers, which limit the kinetics and magnitude of clonal expansion (data not shown), all the activated OTI cells, whether adjuvanted or not, had divided more than the CFSE dilution assay could reliably detect (Figure 3B). However, we did notice that the geometric MFI of adjuvanted OTI cells in WT and TRIF recipients differed and the geometric MFI of non adjuvanted OTI cells did not (Figure 3A and data not shown). Further studies are necessary to conclude the effects of TRIF deficiency on OTI T cell proliferation, but these results suggest that functional TRIF could play a role in TLR4 adjuvant effects on CD8^+^T cell at the level of cellular divisions, as it does for CD4^+^ OTII T cells.

### TRIF deficient mice do not support maximal survival of adjuvanted T cells

TLR4-mediated adjuvant effects on T cells are often seen at the level of increased survival of the cells during or after clonal expansion, an effect that can be modeled ex vivo by culturing explanted cells under growth factor limiting conditions [32], [39]. To determine a role for TRIF in TLR4 mediated effects on activated T cell survival, we activated OTI and OTII cells in WT or TRIF^lps/lps^ recipients in the presence or absence of lipid A. At peak clonal expansion (96 hr) the cells were harvested and cultured ex vivo in the absence of growth factor. Under these conditions antigen stimulated OTI and OTII cells were less viable than non-activated T cells (No Ag Vs. Ag) (Figures 3C & 3D) reflecting the shortened half-lives of T cells activated in the absence of adjuvant. As expected, lipid A treatment during T cell priming significantly improved the ex vivo survival of activated OTI and OTII T cells from WT recipients (Ag+Adj Vs. Ag) (Figure 3C& 3D). The viability of adjuvanted OTI cells from TRIF ^lps/lps^ recipients was low and indistinguishable from non adjuvanted treatments (Figure 3D), indicating that TRIF-dependent signals are critically required for OTI T cell survival. Adjuvanted OTII cells from TRIF ^lps/lps^ mice on the other hand survived better than non-adjuvanted OTII cells but worse than their counterparts from WT mice (Figure 3C), indicating that TRIF deficiency partially affected the OTII survival. These results reveal that OTI and OTII cells are programmed somewhat differently by TLR4 adjuvants to survive growth factor withdrawal, although TRIF was clearly important for both T cell subsets.

### Chemokines influence lipid A adjuvant effects on T cell clonal expansion

**Figure 4:**
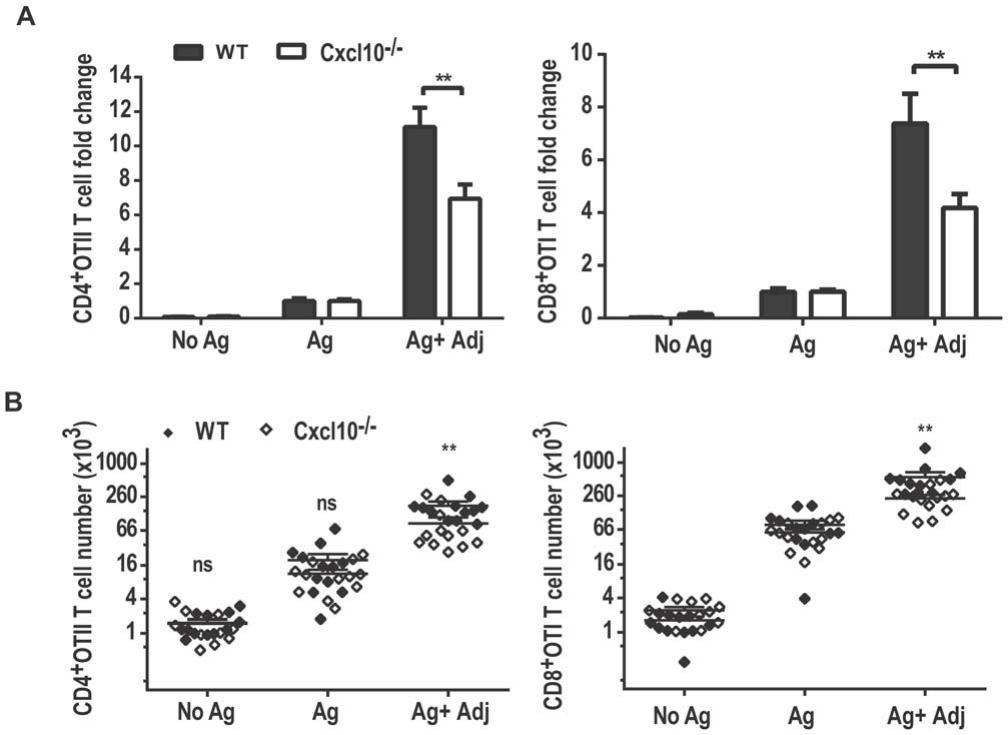
Impaired Lipid A induced adjuvant effects on T cells in CXCLl10^−/−^ recipients. OTI and OTII T cells were activated in WT vs. CXCL10^−/−^ recipients with subcutaneous hock injections containing antigen alone (Ag), antigen plus lipid A (Ag+Adj) or saline (No Ag). Spleens from recipient mice were harvested 96 h after immunization and OTI and OTII T cells were enumerated by flow cytometry. The ratios of OT and OTII cells recovered from the adjuvant treated mice vs. antigen treated mice are plotted as a fold change (A) and in absolute numbers (B). Data are from three independent experiments combining with 3 replicates in each experiment (n = 9). **;P value<0.001.

In addition to cytokines, TLR4 stimulation also induces chemokines, which play an important role in recruiting cells to sites of infection or antigen presentation [40], [41]. CXCL10, also known as IP-10, is a chemokine that is frequently used as a marker for the TRIF-dependent signal upon TLR4 activation [23], [42]. Although CXCL10's role in TLR4-mediated modulation of T cell responses is less clear, earlier reports suggested that CXCL10 promotes the retention of activated T cells in the draining lymph nodes for optimal APC-T cell interactions [41], a role that could explain some or all of the TRIF dependence of TLR4 adjuvant effects on T cells in our experiments. Therefore, we tested whether CXCL10 can influence TLR4-mediated adjuvant effects on T cell clonal expansion. OTI and OTII cell expansion in WT vs. CXCL10^−/−^ recipients was adjuvanted with lipid A and measured by enumerating the cells 96 h later. We found that activated OTI and OTII cells in WT vs. CXCL10^−/−^ recipients expanded to the same extent in the absence of lipid A (Figure 4A), indicating adjuvant-free stimulation of T cells does not require CXCL10. In the presence of lipid A, however, the magnitude of OTI and OTII T cell expansion in response to antigen was significantly smaller in CXCL10^−/−^ recipients as compared to WT. These results indicate that CXCL10, a TRIF-dependent product of TLR4 signaling, contributes to T cell clonal expansion, but is not critically required because significant clonal expansion occurs in its absence.

### Type I IFN signaling is critical for TLR4-mediated adjuvant effects on T cell expansion

**Figure 5:**
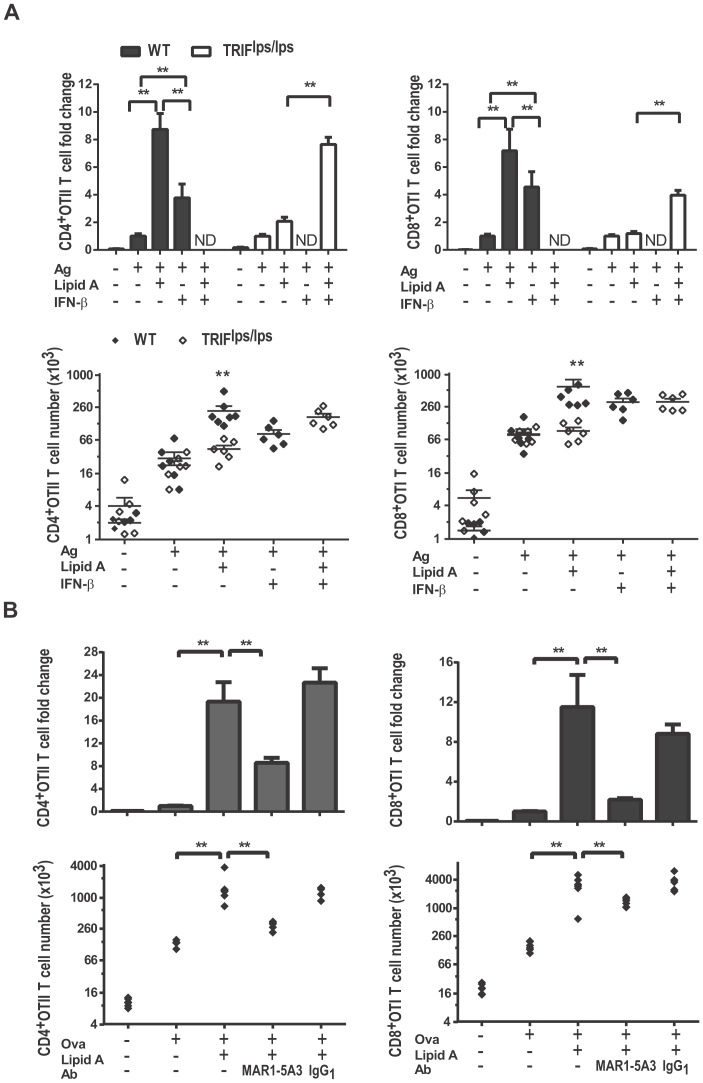
Type I IFN plays an important role in TLR4 mediated adjuvant effects on T cells. OTI and OTII cells were activated in WT or TRIF^lps/lps^ recipients with subcutaneous hock injections containing antigen alone (Ag), with antigen and lipid A (Ag+Adj), or with saline (No Ag). Effects of recombinant IFN-β on T cell adjuvant effects in TRIF^lps/lps^ recipients (A) were analyzed by estimating the lipid A induced fold change in OTI and OT II cell numbers in mice that were infused with 30,000 IU of IFN-β 6,12, and 18 h after immunization over non-adjuvanted treatment. Total OTI or OT II cell numbers observed in spleens of each treated mouse are shown at bottom. Effects of type I IFNα/β receptor blockade (mAb MAR1-5A3)(B) on T cell adjuvant effects in WT recipients was analyzed by estimating the lipid A induced fold change in OTI and OT II cell numbers over non adjuvanted treatment in mice that had been given MAR1-5A3 mAb or control IgG_1_ 1 h before immunization. Total OTI or OT II cell numbers in recipient spleens of each treated mouse at the harvest are shown at bottom. Data shown are pooled from two independent experiments with 3 replicates in each. **; p-value≤0.01 and *; p-value≤0.05. ND; not determined.

Type I IFNs are important cytokines produced upon TLR stimulation, well known for their antimicrobial and immunomodulatory properties. Type I IFN can directly act on DC and T cells to induce cytokines and chemokines that promote growth, survival and interaction of APCs and T cells [43], [44], [45]. Because type I IFN production after TLR4 stimulation is mainly driven by the TRIF signaling branch, we hypothesized that the defective type I IFN production in TRIF ^lps/lps^ mice caused the impaired TLR4 adjuvant effects on T cell clonal expansion. To test this, TRIF^lps/lps^ recipients that received lipid A during immunization were also given 3 doses of recombinant mouse IFN-β (30,000 IU/dose) during the first 18 h after immunization. IFN-β supplementation to TRIF^lps/lps^ mice significantly boosted the peak clonal burst size of both OTI and OTII T cells compared to non-adjuvanted treatments or adjuvanted treatment in TRIF^lps/lps^ recipients (Figure 5A). Similarly, IFN-β administration in place of lipid A to the mice given antigen in WT mice also boosted OTI and OTII cell priming, though to a lesser extent as compared to lipid A treatment (Figure 5A). This partial increase in clonal expansion by IFN-β supplementation compared to lipid A treatment could be due to differences in the IFN-β levels supplemented vs. levels produced in response to lipid A stimulation or could be due to the existence of additional TRIF regulated mechanisms that contribute to the adjuvant effects. To better understand this, we interrupted type I IFN signaling by injecting WT recipient mice with type I IFN receptor blocking monoclonal antibody MAR1-5A3 1 h prior to immunization and measured the T cell clonal expansion 96 h later. We found that MAR1-5A3 blockade reduced the lipid A induced adjuvant effects on OTI T cell expansion by 90% (Figure 5B), while treatment with isotype control antibody had no effect. Interestingly, MAR1-5A3 treatment was less effective in preventing adjuvant effects on OTII T cell clonal expansion; nevertheless a significant reduction compared to adjuvanted OTII cell numbers in WT recipients with intact IFN signal was observed. Together, our results suggest that TRIF-generated type I IFN play an important role in mediating the adjuvant effects of TLR4 agonists on T cells.

**Figure 6:**
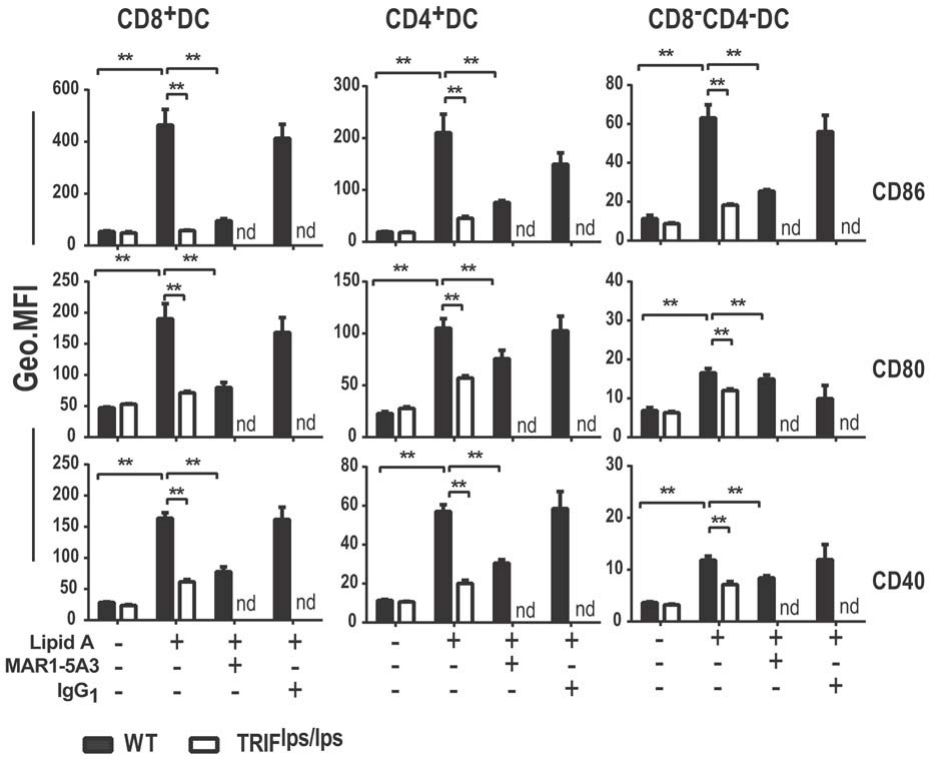
Type I IFN receptor signal is required for TLR4-TRIF mediated upregulation of co-stimulatory molecules in vivo. C57BL/6 and TRIF^lps/lps^ mice were pre-treated with MAR1-5A3 mAb or isotype control and then injected with lipid A (10 µg/mouse) or saline. After 12 h, splenocytes were labeled with fluorochrome conjugated Abs to measure CD80, CD86, and CD40 expression by DC subsets. The data are representative of 2 independent experiments with 3 replicates in each treatment; nd, not determined. Statistical significance between the treatments at each time point was calculated by two-way ANOVA; **; p-value≤0.01 and *; p-value≤0.05 confidence interval.

Earlier studies have shown that type I IFNs play an important role in causing TLR4-induced upregulation of co-stimulatory molecules on the APC [17], an important event that can determine the extent of T cell clonal expansion. In order to determine whether the requirement for type I IFN was at the level of dendritic cell maturation in vivo, WT mice were pre-treated with MAR1-5A3 mAb or isotype control and then treated with lipid A before harvesting the spleen to visualize the maturation of various DC subsets as described in Figure 6. Interfering with the type I IFN signaling in vivo almost abrogated the lipid A-induced upregulation of CD86, CD80, and CD40 on CD8^+^, CD4^+^, and double negative DC populations. The CD86, CD80 and CD40 expression on these DC in the absence of type I IFN signaling was similar to the levels observed in lipid A activated DC in TRIF^lps/lps^ mice (Figure 6). Thus, our results indicate that type I IFNs play a crucial role in TLR4-TRIF-induced upregulation of co-stimulatory molecules on specific DC subsets in vivo.

## Discussion

Natural adjuvants such as LPS and a clinically relevant adjuvant derived from LPS, MPL, modulate adaptive immune responses by influencing early T cell clonal expansion as well as the cytokine milieu expressed during antigen-dependent proliferation by engaging the TLR4/MD2 endotoxin receptor system. However, the contributions of the TLR4 signaling branches that require either MyD88 or TRIF signaling adaptors during initial T cell priming are less clear. In this study, we sought to understand T cell priming events that are modulated by TLR4 agonists in a TRIF-dependent manner. Our results showed that adjuvant effects on the clonal burst size of antigen-specific T cells was severely diminished in TRIF^lps/lps^ mice, indicating the requirement of TRIF-mediated signaling in adjuvanting T cell expansion by TLR4 agonists. Currently available vaccines function primarily by generating protective antibody responses, a process which requires T cell help for B cells. Hence our confirmation that the TRIF pathway is critical in mediating TLR4 adjuvant effects on CD4^+^ T cells is relevant for understanding the improved humoral responses by newly approved adjuvants such as MPL. Generation of stable T cell memory, especially of CD8^+^ CTL, is an ongoing challenge in vaccine design. In this context TRIF may be essential but not sufficient as a mediator of TLR4 adjuvant effects because TRIF is critical for initial clonal expansion, while MyD88 has been reported elsewhere to be needed for maintenance of T cell memory, particularly for CD8^+^ T cells [46].

How does TRIF mediate TLR4 adjuvant effects? Engagement of the TLR4-TRIF signal axis activates interferon response factors (IRFs) that induce type I IFN production. Type I IFN can influence T cell priming by inducing cytokines and chemokines and by directly modulating APC and T cell functions [44], [45], [47]. Hence, we speculated that type I IFN could explain the importance of TRIF for TLR4 adjuvant effects, as has been shown for TLR3 [48]. Consistent with this idea, blockade of type I IFN signaling greatly diminished lipid A's induction of adjuvant effects on both CD8^+^ and, to a lesser extent, on CD4^+^ T cells (Figure 5). Conversely, recombinant IFN β given to TRIF ^lps/lps^ mice during the first 18 hr of T cell priming rescued lipid A-induced adjuvant effects on T cell expansion (Figure 5). Hence type I IFN signaling is indeed critical for TLR4 mediated adjuvant effects on T cell clonal expansion. Type I IFNs are known to play a major role in modulating T cell immune response by TLR3, TLR7, and TLR9 agonists, which are potent inducers of Type I IFN [47], [48], [49], [50]. However, a role for type I IFN in TLR4-mediated adjuvant effects on T cells is not fully characterized. To our knowledge only two other studies have reported a requirement for type I IFN in TLR4-mediated adjuvant effects on T cells, both using IFNAR^−/−^ mice which may have altered APC functions [27], [28]. In the first study, antigen-specific T cell clonal expansion after combined TLR4 and CD40 stimulation was greatly reduced in IFNAR^−/−^ mice compared to WT mice [50]. In the second study, immunization with LPS and antigen failed to generate CTL responses in IFNAR^−/−^ mice [49]. Our study specifically tests for net adjuvant effects, not overall clonal burst size, as a means of isolating the contributions of TLR4, TRIF and type I IFNs to adjuvanticity. Our results together with these earlier reports indicate that type I IFN could be the central players in boosting T cell clonal expansion by TLR4, as well as TLR3, TLR7, and TLR9, agonists.

Earlier studies showed that the TLR4-TRIF-IFN-β axis drives co-stimulatory molecule upregulation in macrophages upon TLR4 stimulation [17]. However, more recent studies reported TRIF-independent upregulation of co-stimulatory molecules on DC upon TLR4 stimulation [18]. Our efforts to clarify contribution of TRIF to DC maturation showed that TLR4/TRIF signaling is sufficient for sustained upregulation of CD86 and CD40 expression on splenic DC. Only CD80 expression required both MyD88 and TRIF signals for maximal expression. Our results may vary from those reported earlier [18] due to differences in the TLR4 agonists used and our analysis of specific DC subsets in an extended time course. Interestingly, as with macrophages, the upregulation of co-stimulatory molecules on DC in vivo was largely mediated by TRIF-dependent type I IFN, because blocking IFN signaling in splenic DC blocked the upregulation of co-stimulatory molecules to the same extent observed in TRIF^lps/lps^ mice. Among different splenic DC subsets, CD8^+^ and CD4^+^ DC showed the biggest changes in the co-stimulatory molecule expression following lipid A stimulation, indicating that they are likely the major players in TLR4-mediated adjuvant effects on T cells. Although B220+ plasmacytoid DCs are potent type I IFN producers, lipid A stimulation had a minimal effect on the maturation of these cells, presumably due to low levels of TLR4 expression by these subsets [51]. It is generally believed that both CD80 and CD86 have similar roles in T cell stimulation. However, it is not clear yet why CD80 but not CD86 require both MyD88 and TRIF and what might be the T cell outcome of more complex regulation of CD80 expression. Some reports show that CD80 signaling on macrophages exacerbates inflammatory responses [52], [53]. Hence, it could be that tighter regulation of CD80 by MyD88 and TRIF is a mechanism for controlled inflammation, or that CD80 is more important for long-term memory responses, specifically those which require both MyD88 and TRIF signals [54].

CXCL10 is a chemokine often used as a signature gene product induced by TLR4 agonists in a TRIF dependent manner [8], [23], [42], [55], although its role in the context of TLR4 stimulation is not clear. Studies have shown that CXCL10 is important for recruitment of T cells and other inflammatory cells to sites of inflammation [40], [56]. In addition, CXCL10 has also been reported to increase APC-T cell interactions by recruiting T cells to APCs [41]. In this study we hypothesized that CXCL10 produced in response to lipid A stimulation could favor enhanced APC-T cell interactions resulting in increased T cell clonal expansion. Consistent with this hypothesis, our results showed that CXCL10 deficiency significantly decreased the magnitude of clonal expansion of both CD4^+^ and CD8^+^ T cells, although the effect was small (less than 2-fold). Type I IFN are important inducers of CXCL10 in response to TLR4 stimulation, although a few studies show existence of TRIF dependent but type I IFN independent mechanisms [8]. Hence, further studies are needed to clarify whether CXCL10 plays a meaningful role in TLR4 adjuvant effects, and if so whether it is a downstream effector of type I IFN signaling, or a TRIF dependent but IFNα/β-independent effector that contributes to TLR4-mediated adjuvant effects on T cell clonal expansion.

TLR4 agonists enhance T cell clonal expansion by boosting both T cell proliferation and survival during proliferation [38]. Our investigations showed that both proliferation and ex vivo survival of CD4^+^ T cells, which predicts T cell maintenance in vivo [39], was significantly affected by TRIF deficiency. Interestingly, ex vivo survival of lipid A-adjuvanted CD8^+^ T cells was completely dependent on functional TRIF, while the effects on CD8^+^ T cells proliferation were inconclusive. TLR-mediated enhancement of T cell proliferation is classically attributed to the upregulation of CD86/CD80 and resulting co-stimulatory signaling. Hence, we speculate that TLR4-TRIF-mediated increases in CD4^+^ T cell proliferation are ultimately due to enhanced co-stimulatory signals, while the differences between CD4^+^ and CD8^+^ T cell proliferation are attributable to intrinsic differences in their proliferative capacities [57]. On the other hand, the precise mechanisms underlying TLR4-TRIF mediated survival signaling to T cells are not clear. Earlier studies showed that type I IFNs can improve the TLR4 induced adjuvanted T cell survival ex vivo [43]. Similarly, other studies showed that the LPS induced activated T cell survival is independent of B7 signals and less dependent on Fas signals or anti-apoptotic proteins, such as Bcl-2 or Bcl-xL induced by co-stimulatory signals [39], [58]. The latter studies suggest that TLR4-TRIF-dependent adjuvanted T cell survival is less dependent on co-stimulatory molecules, although further investigations are necessary for clarification. A more plausible mechanism for TRIF dependency would be the type I IFN induced pathways. The differences in the TRIF requirement for CD4^+^ and CD8^+^ T cell survival could be due to the differences in their responsiveness to the survival signals [59].

Our results highlight the importance of TRIF signaling in the TLR4-mediated adjuvant effects on T cells. TRIF deficient APC responds suboptimally to TLR4 stimulation causing defective DC maturation. In the absence of the TRIF signaling branch TLR4 agonists were impaired in enhancing T cell proliferation as well as their survival. Finally type I IFN plays a central role in the modulation of TLR4/TRIF-mediated events as it does for several other TLRs [48], [49], [50].

## References

[1] Ohashi K, Burkart V, Flohe S, Kolb H (2000) Cutting edge: heat shock protein 60 is a putative endogenous ligand of the toll-like receptor-4 complex. J Immunol 164: 558–561. 10623794 10.4049/jimmunol.164.2.558

[2] Yang D, Postnikov YV, Li Y, Tewary P, de la Rosa G, et al (2012) High-mobility group nucleosome-binding protein 1 acts as an alarmin and is critical for lipopolysaccharide-induced immune responses. J Exp Med 209: 157–171. 22184635 10.1084/jem.20101354 PMC3260868

[3] Palsson-McDermott EM, O'Neill LA (2004) Signal transduction by the lipopolysaccharide receptor, Toll-like receptor-4. Immunology 113: 153–162. 15379975 10.1111/j.1365-2567.2004.01976.x PMC1782563

[4] Chang ZL (2010) Important aspects of Toll-like receptors, ligands and their signaling pathways. Inflamm Res 59: 791–808. 20593217 10.1007/s00011-010-0208-2

[5] Casella CR, Mitchell TC (2008) Putting endotoxin to work for us: monophosphoryl lipid A as a safe and effective vaccine adjuvant. Cell Mol Life Sci 65: 3231–3240. 18668203 10.1007/s00018-008-8228-6 PMC2647720

[6] Hoebe K, Du X, Georgel P, Janssen E, Tabeta K, et al (2003) Identification of Lps2 as a key transducer of MyD88-independent TIR signalling. Nature 424: 743–748. 12872135 10.1038/nature01889

[7] Yamamoto M, Akira S (2010) Lipid A receptor TLR4-mediated signaling pathways. Adv Exp Med Biol 667: 59–68. 20665200 10.1007/978-1-4419-1603-7_6

[8] Weighardt H, Jusek G, Mages J, Lang R, Hoebe K, et al (2004) Identification of a TLR4- and TRIF-dependent activation program of dendritic cells. Eur J Immunol 34: 558–564. 14768061 10.1002/eji.200324714

[9] Kaisho T, Akira S (2002) Toll-like receptors as adjuvant receptors. Biochim Biophys Acta 1589: 1–13. 11909637 10.1016/s0167-4889(01)00182-3

[10] Ulrich JT, Myers KR (1995) Monophosphoryl lipid A as an adjuvant. Past experiences and new directions. Pharm Biotechnol 6: 495–524. 7551233

[11] Alderson MR, McGowan P, Baldridge JR, Probst P (2006) TLR4 agonists as immunomodulatory agents. J Endotoxin Res 12: 313–319. 17059695 10.1179/096805106X118753

[12] Johnson DA (2008) Synthetic TLR4-active glycolipids as vaccine adjuvants and stand-alone immunotherapeutics. Curr Top Med Chem 8: 64–79. 18289078 10.2174/156802608783378882

[13] Garcon N, Van Mechelen M (2011) Recent clinical experience with vaccines using MPL- and QS-21-containing adjuvant systems. Expert Rev Vaccines 10: 471–486. 21506645 10.1586/erv.11.29

[14] Garcon N, Chomez P, Van Mechelen M (2007) GlaxoSmithKline Adjuvant Systems in vaccines: concepts, achievements and perspectives. Expert Rev Vaccines 6: 723–739. 17931153 10.1586/14760584.6.5.723

[15] Fox CB, Friede M, Reed SG, Ireton GC (2010) Synthetic and natural TLR4 agonists as safe and effective vaccine adjuvants. Subcell Biochem 53: 303–321. 20593273 10.1007/978-90-481-9078-2_14

[16] Gelman AE, Zhang J, Choi Y, Turka LA (2004) Toll-like receptor ligands directly promote activated CD4+ T cell survival. J Immunol 172: 6065–6073. 15128790 10.4049/jimmunol.172.10.6065 PMC2833313

[17] Hoebe K, Janssen EM, Kim SO, Alexopoulou L, Flavell RA, et al (2003) Upregulation of costimulatory molecules induced by lipopolysaccharide and double-stranded RNA occurs by Trif-dependent and Trif-independent pathways. Nat Immunol 4: 1223–1229. 14625548 10.1038/ni1010

[18] Shen H, Tesar BM, Walker WE, Goldstein DR (2008) Dual signaling of MyD88 and TRIF is critical for maximal TLR4-induced dendritic cell maturation. J Immunol 181: 1849–1858. 18641322 10.4049/jimmunol.181.3.1849 PMC2507878

[19] Maxwell JR, Ruby C, Kerkvliet NI, Vella AT (2002) Contrasting the roles of costimulation and the natural adjuvant lipopolysaccharide during the induction of T cell immunity. J Immunol 168: 4372–4381. 11970979 10.4049/jimmunol.168.9.4372

[20] McAleer JP, Vella AT (2008) Understanding how lipopolysaccharide impacts CD4 T-cell immunity. Crit Rev Immunol 28: 281–299. 19166381 10.1615/critrevimmunol.v28.i4.20 PMC3549535

[21] McAleer JP, Rossi RJ, Vella AT (2009) Lipopolysaccharide potentiates effector T cell accumulation into nonlymphoid tissues through TRIF. J Immunol 182: 5322–5330. 19380779 10.4049/jimmunol.0803616 PMC2947744

[22] Hou S, Hyland L, Ryan KW, Portner A, Doherty PC (1994) Virus-specific CD8+ T-cell memory determined by clonal burst size. Nature 369: 652–654. 7516039 10.1038/369652a0

[23] Mata-Haro V, Cekic C, Martin M, Chilton PM, Casella CR, et al (2007) The vaccine adjuvant monophosphoryl lipid A as a TRIF-biased agonist of TLR4. Science 316: 1628–1632. 17569868 10.1126/science.1138963

[24] MacKenzie SA, Roher N, Boltana S, Goetz FW (2010) Peptidoglycan, not endotoxin, is the key mediator of cytokine gene expression induced in rainbow trout macrophages by crude LPS. Mol Immunol 47: 1450–1457. 20304498 10.1016/j.molimm.2010.02.009

[25] Fang WF, Cho JH, He Q, Lin MC, Wu CC, et al (2007) Lipid A fraction of LPS induces a discrete MAPK activation in acute lung injury. Am J Physiol Lung Cell Mol Physiol 293: L336–344. 17496062 10.1152/ajplung.00011.2007

[26] Sheehan KC, Lai KS, Dunn GP, Bruce AT, Diamond MS, et al (2006) Blocking monoclonal antibodies specific for mouse IFN-alpha/beta receptor subunit 1 (IFNAR-1) from mice immunized by in vivo hydrodynamic transfection. J Interferon Cytokine Res 26: 804–819. 17115899 10.1089/jir.2006.26.804

[27] Hwang SY, Hertzog PJ, Holland KA, Sumarsono SH, Tymms MJ, et al (1995) A null mutation in the gene encoding a type I interferon receptor component eliminates antiproliferative and antiviral responses to interferons alpha and beta and alters macrophage responses. Proc Natl Acad Sci U S A 92: 11284–11288. 7479980 10.1073/pnas.92.24.11284 PMC40616

[28] Oh JZ, Kurche JS, Burchill MA, Kedl RM (2011) TLR7 enables cross-presentation by multiple dendritic cell subsets through a type I IFN-dependent pathway. Blood 118: 3028–3038. 21813451 10.1182/blood-2011-04-348839 PMC3175780

[29] Hogquist KA, Jameson SC, Heath WR, Howard JL, Bevan MJ, et al (1994) T cell receptor antagonist peptides induce positive selection. Cell 76: 17–27. 8287475 10.1016/0092-8674(94)90169-4

[30] Barnden MJ, Allison J, Heath WR, Carbone FR (1998) Defective TCR expression in transgenic mice constructed using cDNA-based alpha- and beta-chain genes under the control of heterologous regulatory elements. Immunol Cell Biol 76: 34–40. 9553774 10.1046/j.1440-1711.1998.00709.x

[31] Thompson BS, Mitchell TC (2004) Measurement of daughter cell accumulation during lymphocyte proliferation in vivo. J Immunol Methods 295: 79–87. 15627613 10.1016/j.jim.2004.09.008

[32] Sengupta S, Chilton PM, Mitchell TC (2005) Adjuvant-induced survival signaling in clonally expanded T cells is associated with transient increases in pAkt levels and sustained uptake of glucose. Immunobiology 210: 647–659. 16325488 10.1016/j.imbio.2005.06.003

[33] Kamath AT, Pooley J, O'Keeffe MA, Vremec D, Zhan Y, et al (2000) The development, maturation, and turnover rate of mouse spleen dendritic cell populations. J Immunol 165: 6762–6770. 11120796 10.4049/jimmunol.165.12.6762

[34] Asselin-Paturel C, Brizard G, Pin JJ, Briere F, Trinchieri G (2003) Mouse strain differences in plasmacytoid dendritic cell frequency and function revealed by a novel monoclonal antibody. J Immunol 171: 6466–6477. 14662846 10.4049/jimmunol.171.12.6466

[35] Sathe P, Shortman K (2008) The steady-state development of splenic dendritic cells. Mucosal Immunol 1: 425–431. 19079209 10.1038/mi.2008.56

[36] Holt PG, Strickland DH, Wikstrom ME, Jahnsen FL (2008) Regulation of immunological homeostasis in the respiratory tract. Nat Rev Immunol 8: 142–152. 18204469 10.1038/nri2236

[37] Edwards AD, Diebold SS, Slack EM, Tomizawa H, Hemmi H, et al (2003) Toll-like receptor expression in murine DC subsets: lack of TLR7 expression by CD8 alpha+ DC correlates with unresponsiveness to imidazoquinolines. Eur J Immunol 33: 827–833. 12672047 10.1002/eji.200323797

[38] Thompson BS, Mata-Haro V, Casella CR, Mitchell TC (2005) Peptide-stimulated DO11.10 T cells divide well but accumulate poorly in the absence of TLR agonist treatment. Eur J Immunol 35: 3196–3208. 16220541 10.1002/eji.200526132

[39] Vella AT, McCormack JE, Linsley PS, Kappler JW, Marrack P (1995) Lipopolysaccharide interferes with the induction of peripheral T cell death. Immunity 2: 261–270. 7535182 10.1016/1074-7613(95)90050-0

[40] Dufour JH, Dziejman M, Liu MT, Leung JH, Lane TE, et al (2002) IFN-gamma-inducible protein 10 (IP-10; CXCL10)-deficient mice reveal a role for IP-10 in effector T cell generation and trafficking. J Immunol 168: 3195–3204. 11907072 10.4049/jimmunol.168.7.3195

[41] Yoneyama H, Narumi S, Zhang Y, Murai M, Baggiolini M, et al (2002) Pivotal role of dendritic cell-derived CXCL10 in the retention of T helper cell 1 lymphocytes in secondary lymph nodes. J Exp Med 195: 1257–1266. 12021306 10.1084/jem.20011983 PMC2193754

[42] Cekic C, Casella CR, Eaves CA, Matsuzawa A, Ichijo H, et al (2009) Selective activation of the p38 MAPK pathway by synthetic monophosphoryl lipid A. J Biol Chem 284: 31982–31991. 19759006 10.1074/jbc.M109.046383 PMC2797270

[43] Marrack P, Kappler J, Mitchell T (1999) Type I interferons keep activated T cells alive. J Exp Med 189: 521–530. 9927514 10.1084/jem.189.3.521 PMC2192920

[44] Curtsinger JM, Valenzuela JO, Agarwal P, Lins D, Mescher MF (2005) Type I IFNs provide a third signal to CD8 T cells to stimulate clonal expansion and differentiation. J Immunol 174: 4465–4469. 15814665 10.4049/jimmunol.174.8.4465

[45] Gonzalez-Navajas JM, Lee J, David M, Raz E (2012) Immunomodulatory functions of type I interferons. Nat Rev Immunol 12: 125–135. 22222875 10.1038/nri3133 PMC3727154

[46] McAleer JP, Zammit DJ, Lefrancois L, Rossi RJ, Vella AT (2007) The lipopolysaccharide adjuvant effect on T cells relies on nonoverlapping contributions from the MyD88 pathway and CD11c+ cells. J Immunol 179: 6524–6535. 17982041 10.4049/jimmunol.179.10.6524

[47] Havenar-Daughton C, Kolumam GA, Murali-Krishna K (2006) Cutting Edge: The direct action of type I IFN on CD4 T cells is critical for sustaining clonal expansion in response to a viral but not a bacterial infection. J Immunol 176: 3315–3319. 16517698 10.4049/jimmunol.176.6.3315

[48] Longhi MP, Trumpfheller C, Idoyaga J, Caskey M, Matos I, et al (2009) Dendritic cells require a systemic type I interferon response to mature and induce CD4+ Th1 immunity with poly IC as adjuvant. J Exp Med 206: 1589–1602. 19564349 10.1084/jem.20090247 PMC2715098

[49] Durand V, Wong SY, Tough DF, Le Bon A (2004) Shaping of adaptive immune responses to soluble proteins by TLR agonists: a role for IFN-alpha/beta. Immunol Cell Biol 82: 596–602. 15550117 10.1111/j.0818-9641.2004.01285.x

[50] Ahonen CL, Doxsee CL, McGurran SM, Riter TR, Wade WF, et al (2004) Combined TLR and CD40 triggering induces potent CD8+ T cell expansion with variable dependence on type I IFN. J Exp Med 199: 775–784. 15007094 10.1084/jem.20031591 PMC2212721

[51] Kadowaki N, Ho S, Antonenko S, Malefyt RW, Kastelein RA, et al (2001) Subsets of human dendritic cell precursors express different toll-like receptors and respond to different microbial antigens. J Exp Med 194: 863–869. 11561001 10.1084/jem.194.6.863 PMC2195968

[52] Eri R, Kodumudi KN, Summerlin DJ, Srinivasan M (2008) Suppression of colon inflammation by CD80 blockade: evaluation in two murine models of inflammatory bowel disease. Inflamm Bowel Dis 14: 458–470. 18186109 10.1002/ibd.20344

[53] Nolan A, Kobayashi H, Naveed B, Kelly A, Hoshino Y, et al (2009) Differential role for CD80 and CD86 in the regulation of the innate immune response in murine polymicrobial sepsis. PLoS One 4: e6600. 19672303 10.1371/journal.pone.0006600 PMC2719911

[54] Pasare C, Medzhitov R (2004) Toll-dependent control mechanisms of CD4 T cell activation. Immunity 21: 733–741. 15539158 10.1016/j.immuni.2004.10.006

[55] Fitzgerald KA, Rowe DC, Barnes BJ, Caffrey DR, Visintin A, et al (2003) LPS-TLR4 signaling to IRF-3/7 and NF-kappaB involves the toll adapters TRAM and TRIF. J Exp Med 198: 1043–1055. 14517278 10.1084/jem.20031023 PMC2194210

[56] Liu M, Guo S, Hibbert JM, Jain V, Singh N, et al (2011) CXCL10/IP-10 in infectious diseases pathogenesis and potential therapeutic implications. Cytokine Growth Factor Rev 22: 121–130. 21802343 10.1016/j.cytogfr.2011.06.001 PMC3203691

[57] Foulds KE, Zenewicz LA, Shedlock DJ, Jiang J, Troy AE, et al (2002) Cutting edge: CD4 and CD8 T cells are intrinsically different in their proliferative responses. J Immunol 168: 1528–1532. 11823476 10.4049/jimmunol.168.4.1528

[58] Mitchell TC, Teague TK, Hildeman DA, Bender J, Rees WA, et al (2002) Stronger correlation of bcl-3 than bcl-2, bcl-xL, costimulation, or antioxidants with adjuvant-induced T cell survival. Ann N Y Acad Sci 975: 114–131. 12538159 10.1111/j.1749-6632.2002.tb05946.x

[59] Saibil SD, Jones RG, Deenick EK, Liadis N, Elford AR, et al (2007) CD4+ and CD8+ T cell survival is regulated differentially by protein kinase Ctheta, c-Rel, and protein kinase B. J Immunol 178: 2932–2939. 17312138 10.4049/jimmunol.178.5.2932

